# Nerves and nodules: Mononeuritis multiplex as a reversible complication of severe Hidradenitis Suppurativa

**DOI:** 10.1016/j.jdcr.2026.01.054

**Published:** 2026-02-09

**Authors:** Isaree Pitaktong, Om Patel, David S. Sandlin, Lauren A.V. Orenstein, Marissa L.H. Baranowski

**Affiliations:** a Department of Dermatology, Emory University School of Medicine, Atlanta, Georgia; b Department of Internal Medicine, Vanderbilt University Medical Center, Nashville, Tennessee; c Department of Neurology, Emory University School of Medicine, Atlanta, Georgia

**Keywords:** Hidradenitis Suppurativa, mononeuritis multiplex, multifocal neuropathy

## Introduction

Mononeuritis Multiplex (MM) is a peripheral neuropathy marked by asynchronous, multifocal ischemic lesions of non-contiguous peripheral nerves, causing acute or subacute pain, sensory loss, and weakness in discrete nerve distributions.^1^ Hidradenitis Suppurativa (HS) is a chronic inflammatory skin disorder involving recurrent painful flares, central sensitization, and altered peripheral nerve function.^2^ About 30% of HS patients experience neuropathic pain,^3^ yet the co-occurrence of HS and MM is rare. To our knowledge, only 1 prior report has described an HS patient developing neurological weakness. A patient with HS complicated by squamous cell carcinoma developed subacute muscle weakness and sensory symptoms, which resolved following tumor and HS excision.^4^ Thus, a link between HS and MM remains largely unexplored.

## Case report

**Fig 1:**
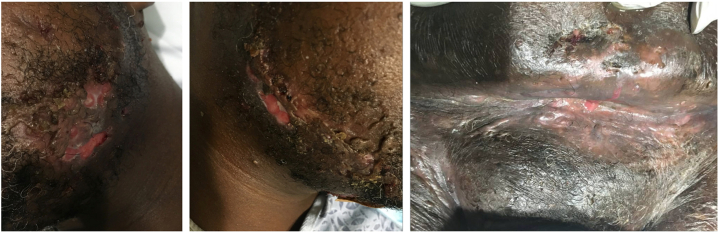
Initial hospital admission. From left to right: left jawline, right jawline, pannus and mons pubis.

A 44-year-old male with an over 10-year history of HS was admitted for worsening disease with 2 weeks of severe burning, shooting pain in the left arm and leg that prevented ambulation. Pain was 10/10 and involved the left hand, wrist, elbow, foot, and ankle, worsened by pressure and relieved with rest, with associated left ulnar paresthesia. Concurrently, he noticed new abscess formation that progressed to purulent drainage from a baseline of no open or draining lesions. Medical history included class 2 obesity, prediabetes, and daily tobacco use (11 pack-years). Prior HS excisions involved the left axilla, bilateral groin, and sacrum, most recently 5 years earlier. Family history included fibromyalgia and lupus. Skin exam revealed extensive draining tunnels and indurated nodules consistent with Hurley Stage III HS affecting the mandible, pannus, and groin (Fig 1). Neurological exam demonstrated dysesthesia and diminished light-touch sensation in left digits 4 and 5 without weakness or reflex abnormalities. Brain magnetic resonance imaging (MRI) was unremarkable. Computed tomography imaging of the abdomen and pelvis (CT A/P) confirmed sinus tracts without drainable abscess, and blood cultures were negative. Neurology considered MM versus musculoskeletal pain. Leukocytosis and pain improved with vancomycin, cefazolin, gabapentin, ibuprofen, and oxycodone. The patient was discharged on doxycycline 100 mg twice daily (BID) and gabapentin 1200 mg three times a day (TID) with ambulatory assistance.

**Fig 2:**
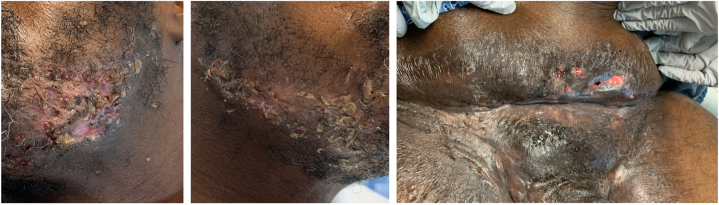
Hospital admission 2 months after initial admission. From left to right: left jawline, right jawline, pannus and mons pubis.

Two months later, the patient was readmitted for severe shooting midline low back pain and weakness preventing ambulation. The pain was sharp, continuous, rated 8/10, worsened with weight-bearing and unresponsive to gabapentin and cyclobenzaprine. Left-sided arm and leg pain from the prior admission had partially receded, persisting at the left ulnar hand with pain 5-6/10. Hurley Stage III HS lesions affecting the mandible and inguinal area persisted with purulent drainage (Fig 2). Neurologic examination revealed expanded sensory deficits involving diminished pinprick sensation in left thumb and digits 4 and 5, left forearm, right medial forearm, and left lateral heel. Motor exam demonstrated asymmetric left upper extremity weakness (4-4+/5 strength) in the shoulder abductors, elbow flexors, wrist extensors, triceps, finger abductors, and abductor pollicis brevis, with left forearm atrophy and reduced left achilles reflex. Leukocytosis and C-Reactive Protein were markedly increased from the previous admission. Extensive laboratory evaluation for vasculitis and connective tissue disease was unrevealing. Blood cultures remained negative. CT A/P demonstrated new sinus tract formation without drainable abscess. MRI of the lumbar spine showed mild T12-L1 and L4-L5 interspinous ligament inflammation without myositis, and electromyography/nerve conduction (EMG/NCS) studies of the LUE were unremarkable. Given concern for worsening infection, empiric vancomycin and cefepime were initiated. Doxycycline was replaced with clindamycin 300 mg BID and rifampin 600 mg daily due to limited HS response. Gabapentin was replaced with pregabalin 50 mg BID due to lack of neuropathic improvement. Ibuprofen and oxycodone were provided. During hospitalization, leukocytosis and neuropathic pain improved, back pain decreased to 5-6/10 and mobility increased. The patient was discharged on clindamycin, rifampin, pregabalin, and oxycodone.

Over the next 2 months, while awaiting financial sponsorship for biologic therapy, the patient was hospitalized twice for severe back pain and recurrent draining HS lesions. He reported paresthesia over the thenar eminences and developed sensory loss affecting the bilateral upper extremities and left lower extremity, distal worse than proximal and left worse than right. Left upper extremity weakness persisted. Treatment included 2 4-week prednisone tapers beginning at 60 mg daily, interspersed by an L4-L5 epidural steroid injection, each providing transient pain relief. Pregabalin was escalated to 100 mg BID, duloxetine was initiated and escalated to 60 mg daily, and opioid therapy continued.

**Fig 3:**
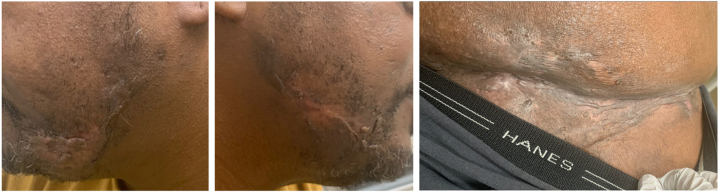
Office visit 6 months after initial admission, after 2 doses of infliximab. From left to right: left jawline, right jawline, pannus and mons pubis.

After sponsorship approval, infliximab was initiated at 10 mg/kg intravenously with standard induction at weeks 0, 2, and 6 and 4-week maintenance dosing. Methotrexate 7.5 mg weekly was added to reduce anti-drug antibody formation. After 2 induction doses, the patient experienced marked remission of HS in multiple areas (Fig 3) with complete resolution of neuropathic pain and motor strength returned to 5/5 bilaterally except for a residual left grip weakness (4/5). He has remained on infliximab at the same dose and frequency for 4 years with sustained HS control, complete resolution of weakness, and reversal of muscular atrophy. A 4-month treatment lapse due to a loss of sponsorship 3 years after treatment initiation resulted in an HS flare with painful draining lesions on the groin and buttocks and mild recurrence of left-sided weakness without atrophy, both resolving after infliximab re-initiation.

## Discussion

HS has been associated with neuropathic pain^3^ and peripheral neuropathy,^5^ and MM most commonly occurs in the setting of systemic inflammatory disease.^6^ In this patient, acute distal multifocal neuropathic pain followed by asymmetric weakness, muscle atrophy and reflex changes during severe HS flares supports an inflammatory neuropathy suggestive of MM associated with active HS. Notably, neurological symptoms closely paralleled HS activity, resolving with immunomodulatory therapy and emerging during treatment lapses. Given that both HS and MM are immune-related pathologies, the immunomodulation and effective HS control likely reduced systemic inflammation and facilitated resolution of neurological symptoms. Although limited by a clinical diagnosis of MM with normal EMG/NCS findings and absence of nerve biopsy, this temporal association suggests MM may represent a rare systemic inflammatory complication of HS. Clinicians should consider inflammatory neuropathy in patients with severe or uncontrolled HS to facilitate early detection and treatment.

## Conflicts of interest

Orenstein received personal consulting fees from Novartis, UCB, and Incyte; served as an investigator with Novartis; received a grant from Pfizer; served as an Associate Editor for *Dermatology*; and was a member of the Board for the HS Foundation. Baranowski participated in an advisory board for UCB and served as a consultant for ExpertConnect.
